# PD144 Uncertainty In Precision Medicine: The Value Of Research To Support The Value Of Personalized Intervention Policies

**DOI:** 10.1017/S0266462324003799

**Published:** 2025-01-07

**Authors:** Claire Rothery, Fernando Alarid Escudero, Natalia Kunst, Andrea Manca

## Abstract

**Introduction:**

One-size-fits-all policies are not always optimal. Stratified decision-making is only possible when the characteristics used to define subgroups can be identified for stratum-specific predictions of outcomes. Conditioning decisions on characteristics that are not readily known may require information (diagnostic, prognostic, predictive) to be derived, the value of which needs to be assessed to support personalized strategies.

**Methods:**

A general framework was developed to show how personalized policies can be accountably informed by characterizing uncertainty, heterogeneity, and bias in evidence. In the framework, observed heterogeneity was disentangled from random variability by conditioning the value of the model input parameters on a set of prognostic or predictive variables, while unobserved heterogeneity was quantified as the systematic variability that cannot be explained given current information. Value of information analysis was used to quantify the value of additional information for resolving decision uncertainty in model input parameters and to identify individual- or subgroup-level attributes that contribute to the degree of heterogeneity.

**Results:**

Decision-making based on average cost effectiveness fails to account for the role that sources of outcome variability play in guiding nuanced decision-making. Conditioning on a set of known covariates to reflect observable heterogeneity may be extended to conditioning on the latent random variable for unobservable covariates to quantify unobservable heterogeneity. Quantifying the potential value of research to inform subgroup- or individual-level attributes may be used to direct further research toward the attributes expected to be of most interest because they drive the value of individualized decisions—the expected value of sample information for attributes.

**Conclusions:**

Two distinct, but interrelated concepts for assessing the value of stratified decision-making are important: (i) the value of heterogeneity; and (ii) the value of further research to inform both heterogeneous factors and to reduce decision uncertainty in precision medicine. Assessing the value of unexplained heterogeneity and bias can be central to supporting the value of personalized intervention strategies in health technology assessment.

